# Jérôme Lejeune (1926-1994): A Pioneer in Uncovering the Connection Between Congenital Conditions and Chromosomal Anomalies

**DOI:** 10.7759/cureus.75643

**Published:** 2024-12-13

**Authors:** Ilaria Bertini, Costanza Raimondi

**Affiliations:** 1 Law and Medical Ethics, Bios Centre, London, GBR; 2 Healthcare Surveillance and Bioethics, Università Cattolica del Sacro Cuore, Rome, ITA

**Keywords:** chromosomal anomaly, chromosomes, cytogenetics, disability, genetics, nuclear radiation, stigma, trisomy 21

## Abstract

Jérôme Lejeune was a French physician and geneticist whose crucial contribution to the field of medicine was the discovery of an extra copy of chromosome 21 in those presenting with a range of physical and developmental anomalies known as Down syndrome. From this discovery on, the condition had a new name (trisomy 21) and a specific scientific explanation that left no room for discrimination against those affected and their parents. Lejeune promoted the idea that a medical doctor should hate the condition and love the patient: while working to find a cure for trisomy 21, Lejeune was also able to reassure his patients and their families and lead them out from under a long-standing stigma inflicted upon them. He was also considered an expert in nuclear radiation and its effects on human genetic material.

## Introduction and background

The aim of this article is to highlight the pivotal contributions of Jérôme Lejeune to genetics, particularly cytogenetics. Lejeune was a French pediatrician and geneticist who began his career working with children with Down syndrome under the supervision of Professor Raymond Turpin. Concurrently, he also had an interest in nuclear radiation, concerning the potential effects on human genetic material. He became an internationally recognized expert also in this field.

**Figure 1:**
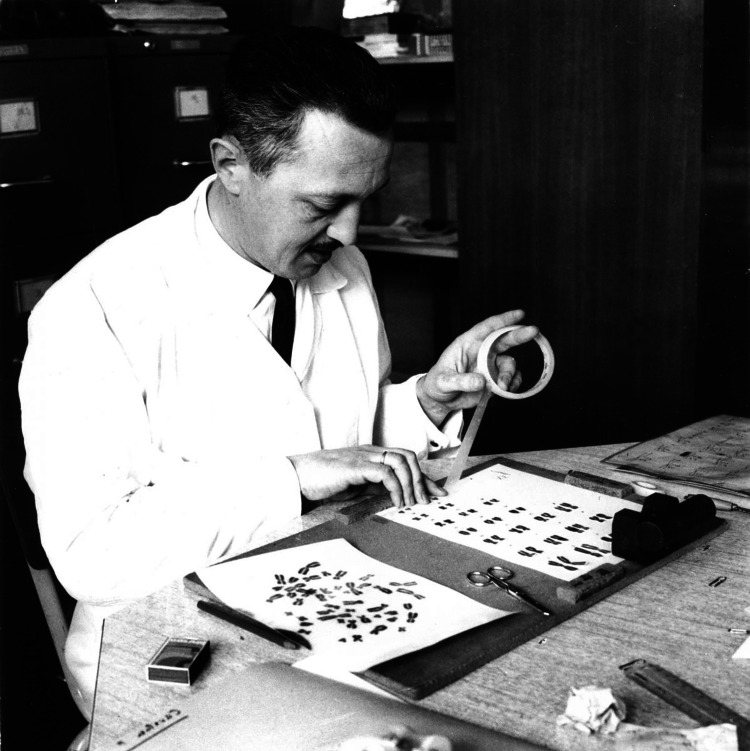
Jérôme Lejeune working on the hypothesis of an extra chromosome in patients with Down syndrome Reference: [1]

**Figure 2:**
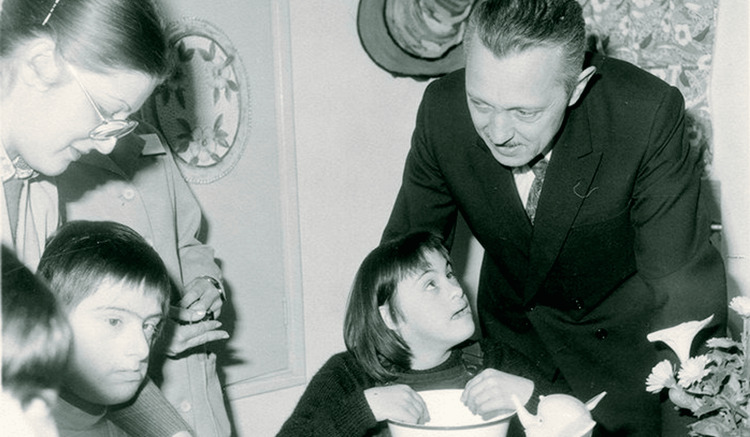
Jérôme Lejeune with some of his young patients Reference: [1]

In 1956, following the discovery by Levan and Tjio that human somatic cells contain 46 chromosomes, Lejeune pursued the hypothesis that Down syndrome was caused by a chromosomal irregularity (Figure 1) [1]. In order to do so, it was crucial the unique cell culture technique brought back to France from the United States by colleague Martha Gautier [2]. He confirmed this hypothesis in 1958 by identifying an extra chromosome in the cells of children with the syndrome, also known as trisomy 21 [3]. This ground-breaking discovery opened a new chapter for cytogenetics and laid the foundation for identifying many other syndromes related to chromosomal anomalies [3]. His unique contribution also came from the unity he perceived between research activity and clinical activity, which he carried out jointly throughout his entire career with inspiring dedication and passion (Figure 2) [4,1].

## Review

Lejeune's life and career

Born in 1926 in Montrouge, a south Parisian suburb, Jérôme Lejeune was the second son of Marguerite Marcelle Lermat and Pierre-Ulysse Lejeune. Lejeune studied medicine and began his medical career working with children with Down syndrome under the supervision of Professor Raymond Turpin, who was the head of the Pediatrics Unit at Armand-Trousseau Hospital in Paris, with the aim of discovering the cause of mental disability [5]. He started his journey as a research intern at the French National Centre for Scientific Research (CNRS) in 1952 studying nuclear radiation and its effects on human genetic material and quickly advanced by 1956 to head of research. In the meantime, he earned a degree in genetics in 1954 and in biochemistry in 1955.

After the atomic bombing of Hiroshima and Nagasaki, Lejeune started working on the effects of radiation on human genes. His first relevant publication, coauthored with Turpin, was about the impact of ionic radiations on the stability of the human hereditary patrimony in particular the higher risk to develop cancer and to become sterile [6].

In 1957, Lejeune was also appointed as a French expert on the United Nations Scientific Committee on the Effects of Atomic Radiation [4,5].

In 1958, Lejeune discovered the cause of Down syndrome as connected to a chromosomal irregularity, specifically the presence of an extra copy of chromosome 21. In 1962, his extraordinary discovery, which for the first time linked a condition of mental disability to a chromosomal anomaly, was honored with the Kennedy Prize, awarded to him personally by President John F. Kennedy. Following this discovery, Lejeune, in collaboration with various colleagues, uncovered the mechanisms behind other chromosomal disorders, thereby opening a new chapter in the discipline of cytogenetics [4,5].

In 1964, Lejeune was promoted to Professor of Fundamental Genetics at the Faculty of Medicine in Paris: he was the first person to hold this title, as the position was specifically created for him. In the same year, he became head of the Cytogenetics Unit at the Necker-Enfants Malades Hospital [7]. Lejeune also received the Allen Memorial Award, the most important prize for genetics which was presented to him at the annual meeting of the American Society of Human Genetics in 1969 [5,7].

**Figure 3:**
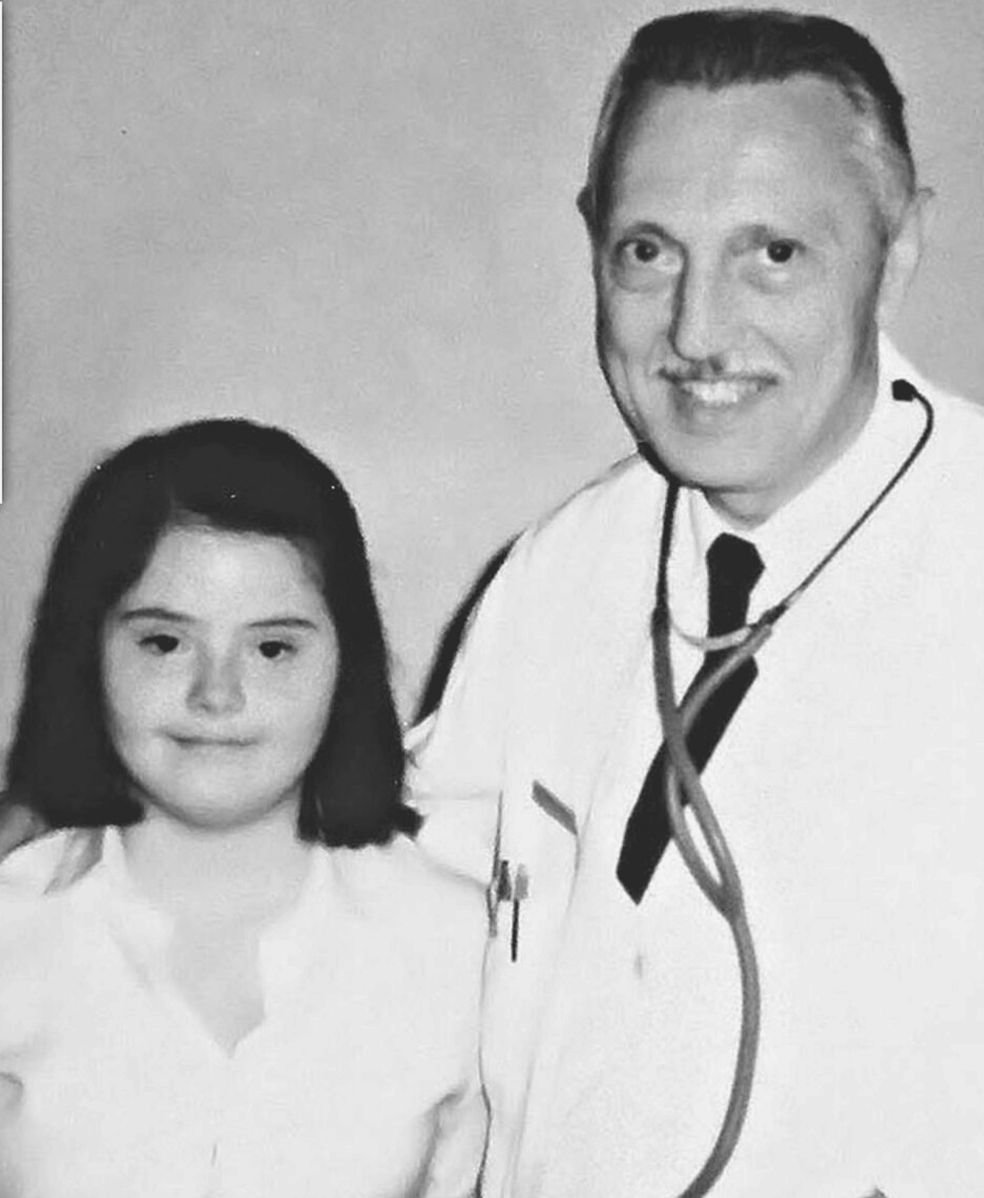
Jérôme Lejeune with one of his young patients Reference: [1]

In the years following his discoveries, he traveled around the world for conferences and to offer expert opinions. He also became an advocate for those affected by Down syndrome, who had suffered from a huge stigma because of their condition. Their unique physical features led to the mistaken belief that they were linked to the people of Mongolia. This is why Sir Langdon Down coined the term "mongolism" in 1866, referring to it as Mongolian idiocy. However, this racist explanation was clearly inadequate. As has happened many times in human history, in the absence of an apparent explanation, a cloud of shame fell over the parents and the disabled children [5,8]. Throughout his career, Lejeune received numerous proposals to collaborate with other universities and laboratories, which he always declined in order to remain in Paris, close to his patients and their families (Figure 3) [1]. Lejeune passed away from lung cancer at the age of 67, leaving behind his wife Birthe, their five children, and numerous grandchildren [5,8].

The opening of a new chapter for cytogenetics: the discovery of trisomy 21

In the late 1800s, the English physician Sir Langdon Down (1828-1896) became the chief physician of the largest "Asylum for Idiots" in England, the so-called Earlswood Asylum, where he started to study the somatic features of mentally retarded people. Borrowing Johann Blumenbach's classification of races, he described the largest family group in the Earlswood Asylum as part of the Mongolian family: the mongoloid idiots. Children born with these traits were typically institutionalized, and in the rare cases where a family chose not to abandon their child, they were left to manage on their own. With little understanding of the causes behind these mental disabilities or whether they were hereditary, families faced significant stigma [9].

The discovery of Levan and Tjio in 1956, according to which normal human somatic cells contain 46 chromosomes, laid the foundation for Lejeune and his colleagues to unveil the reason for mental disability in children with Down syndrome. The karyotype technique developed by Levan and Tjio, which improved the chromosomes' image, shortening the pre-treatment in the hypotonic solution and adding a colchicine dose to the tissue cultures made their counting more accurate [10]. However, the discovery of Levan and Tjio came only from the analysis of the somatic tissue of aborted embryos lacking and then leaving unaccounted the chromosome number in the sex cells, which was discovered a few months later in 1956 by Ford and Hamerton [11]. Lejeune's research started by comparing the skin fibroblasts of monozygotic and heterozygotic twins as a straightforward way to establish the genetic component of a congenital disorder [11,12]. It was then noted that the concordance between monozygotic twins suggested that the determining lesion occurred early in the embryo's development (before the 15th day), indicating a genetic mechanism [11].

**Figure 4:**
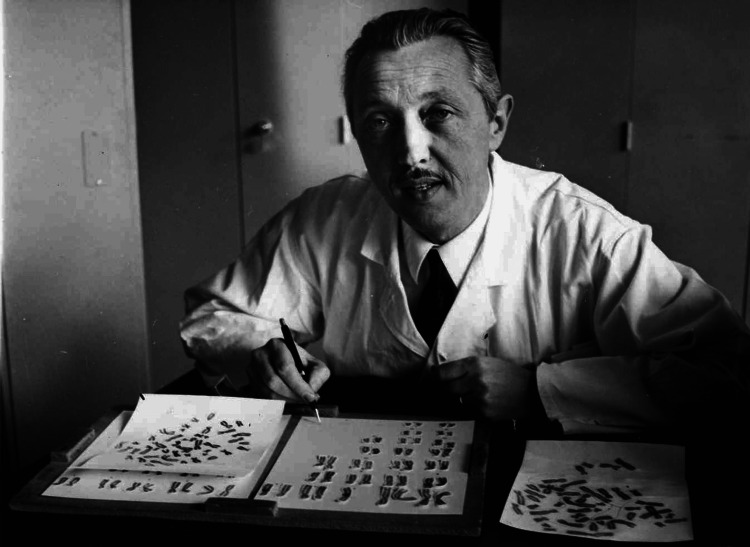
Jérôme Lejeune in his laboratory cutting photographs and pairing the chromosomes of one of his patients Reference: [1]

Lejeune understood that the problem must have been at the chromosomal level, and therefore, it became crucial to count the chromosomes, which he was able to do, thanks to the new tissue culture technique imported from the United States by his colleague Gautier [2]. Perfectioning this new technique, Lejeune and his team managed to intensely stain the chromosomes, so that it was more manageable to count them and take pictures to have proof of their findings (Figure 4) [1]. In January 1959, after two years of trials and errors, and after some doubts, Lejeune, Gautier, and Turpin published their first note to the French Academy of Sciences in which they stated that they had observed an extra chromosome in six children with Down syndrome [4]. In March of the same year, a study concerning nine children with Down syndrome was published [13] and finally stated that the cause of such a condition must be the extra chromosome [14]. In particular, the karyotype analysis of five boys and four girls showed a small telocentric extra chromosome (six instead of five in normal men and five instead of four in normal women) which explains the abnormal figure of 47 [12].

Other researchers around the world soon started to support these findings. There were some doubts about whether the anomaly involved chromosome 21 or 22, but eventually, it was confirmed that it was trisomy 21 [11]. This meant that Down syndrome resulted in an excess of genes in the affected individual. Later, new experimental observations have identified the rare occurrence of trisomy 21 with 46 chromosomes, which can result from either a translocation, where a full or partial copy of chromosome 21 attaches to another chromosome, or mosaicism, where some cells contain 46 chromosomes while others have 47 [11].

The discovery of trisomy 21 not only provided a scientific explanation for the intellectual disabilities associated with Down syndrome, making the outdated term "Mongolian idiots" obsolete, but it also helped to dispel the stigma that had been placed on families due to the previously unexplained nature of the condition. However, "environmental factors," such as maternal age that could statistically increase the risk of this chromosome pathology, were not ruled out in the light of their experimental evidence [11]. 

The discovery of cri-du-chat syndrome

The years immediately following the discovery of trisomy 21 were exceptional for the identification of new chromosome pathologies such as trisomy 13 and 18 syndromes and many sex chromosome syndromes due to various aberrations (translocations, deletions, etc.) [11].

In 1963, Lejeune and his team discovered cri-du-chat syndrome. Patients with this pathology present with learning disability and physical anomalies including a cry that echoes the miaowing of a cat [11,15]. The condition was found to be linked to a chromosomal deletion: loss of about half the length of the short arm of one copy of chromosome 5 [11,15,16]. Thanks to the discoveries of Lejeune and his team, a new approach to the study of the origin of various diseases and conditions started to take place [8]; for instance, the causes of what came to be called Turner syndrome were discovered soon after [5,11].

## Conclusions

Lejeune's discovery of trisomy 21 paved the way for the study of human cytogenetics and the identification of numerous other chromosomal anomalies. Lejeune's relentless pursuit of a cure for trisomy 21 not only intensified his research efforts but also heightened his empathy for his patients and their families. His position vis-à-vis his patients was to hate the condition and love the patient: while recognizing the limitations and struggles that trisomy 21 imposed on those affected, and in a ceaseless effort to cure them, Lejeune was still able to affirm the dignity of their life and to embrace them as patients who needed to be seen and cared for. In the disability community, Lejeune's discovery and efforts to find a cure were sometimes interpreted as implying that individuals with Down syndrome were not acceptable as they are, but if you look at his life, it is clear that he fought to emphasize their inherent value first of all while addressing the medical challenges they face. In a medical culture often so fragmented, the life of Lejeune shows that it is possible to be deeply scientific and profoundly human.
